# P-716. Limit of Detection and Inclusivity of a Research-Use-Only (RUO) Prototype of a Multiplexed Sample-to-Answer PCR System for the Identification of Vaginal Infections from Vaginal Swabs

**DOI:** 10.1093/ofid/ofaf695.928

**Published:** 2026-01-11

**Authors:** Angela Clark, Brandon Hanberg, Ashley Hillman, Crystal Smith, Jessica Papenfuss, Craig Chandler, Robert Trauscht, J Nicholas Francis, Michael Vaughn, Justin Keener, Usha Spaulding, Margarita Rogatcheva

**Affiliations:** bioMérieux, Salt Lake City, UT; bioMérieux, Salt Lake City, UT; bioMérieux, Salt Lake City, UT; bioMérieux, Salt Lake City, UT; bioMérieux, Salt Lake City, UT; bioMérieux, Salt Lake City, UT; bioMérieux, Salt Lake City, UT; bioMérieux, Salt Lake City, UT; bioMérieux, Salt Lake City, UT; bioMérieux, Salt Lake City, UT; bioMérieux, Salt Lake City, UT; bioMérieux, Salt Lake City, UT

## Abstract

**Background:**

Bacterial vaginosis (BV), vulvovaginal candidiasis (VVC), and trichomoniasis, caused by *Trichomonas vaginalis,* are prevalent, symptomatically similar conditions causing vaginal discharge, discomfort, and adverse reproductive outcomes, if left untreated.

bioMérieux is developing the BIOFIRE® SPOTFIRE® Vaginitis (VG) Panel, a syndromic, molecular test designed for the point-of-care setting to test a vaginal swab (Vswab) in eNAT® media, providing rapid diagnosis (≤ 20 minutes) for BV, VVC (specification of six *Candida* species), and trichomoniasis. These study results detail the limit of detection (LoD) and reactivity of the RUO Panel assays.

**Figure:**
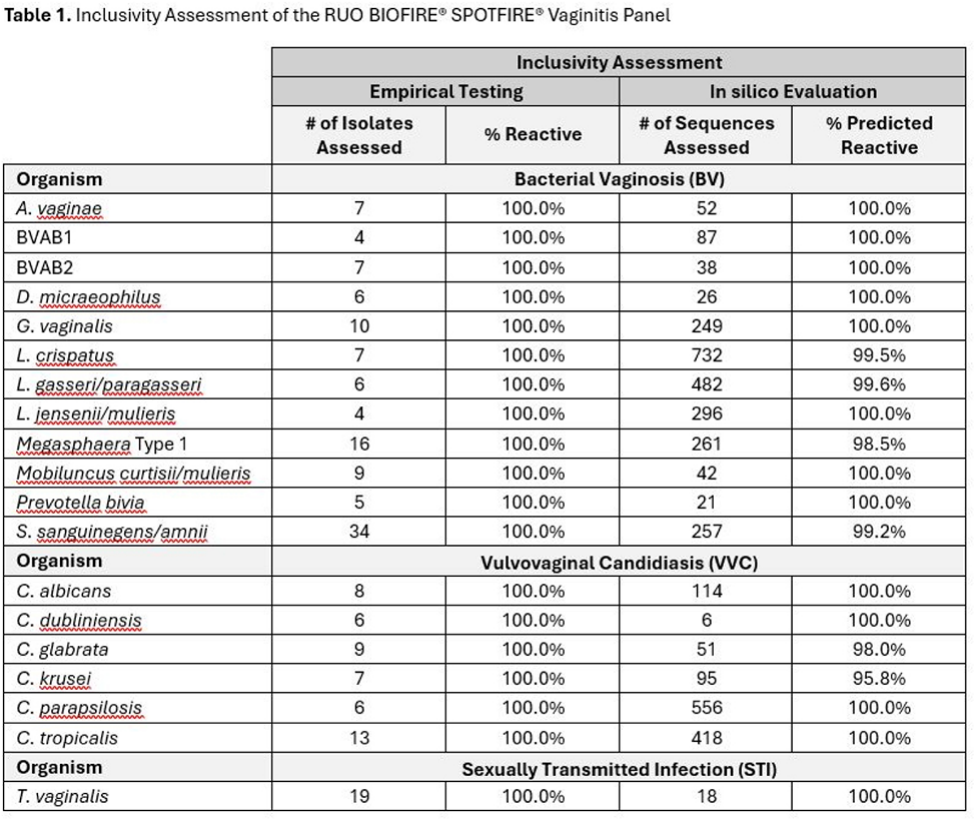

**Methods:**

95% LoD was determined for panel analytes using a dilution series of molecularly quantified strains in sample collection medium (eNAT) in the presence and absence of Vswab matrices. Ten unique Vswab samples were tested to evaluate the impact of matrix on the LoD of panel assays. Inclusivity was empirically assessed by testing 183 isolates, or synthetic nucleic acids (for unculturable or inaccessible analytes) at 3X – 10X LoD. Additionally, in silico assessments were performed to determine the reactivity of specific assay primers to > 3800 sequences representing agents of BV, VVC and trichomoniasis.

**Results:**

The LoD ranged from 4.19E+01 to 5.93E+03 copies/mL for BV analytes, from 1.18E+02 to 1.05E+04 copies/mL for *Candida* species and 5.75E-01 copies/mL for *T. vaginalis* in Vswab. The LoD in Vswab was equal to or within 10X of eNAT demonstrating robust detection across multiple Vswab samples. See Table 1 for a summary of inclusivity. No limitation to reactivity was observed for 183/183 isolates tested. In silico analysis predicted 99.7% of available sequences to be reactive with panel assays.

**Conclusion:**

These results indicate that the BIOFIRE® SPOTFIRE® VG Panel can correctly identify key pathogens implicated in vaginitis from vaginal swab samples with a high degree of accuracy.

This abstract contains data regarding a device that has not been reviewed or approved by regulatory agencies for in vitro diagnostic use.

**Disclosures:**

All Authors: No reported disclosures

